# Transfer of the small diatoms *Thalassiosira proschkinae* and *T*. *spinulata* to the genus *Minidiscus* and their taxonomic re-description

**DOI:** 10.1371/journal.pone.0181980

**Published:** 2017-09-05

**Authors:** Joon Sang Park, Seung Won Jung, Jang-Seu Ki, Ruoyu Guo, Hyun Jung Kim, Kyun-Woo Lee, Jin Hwan Lee

**Affiliations:** 1 Department of Life Science, Sangmyung University, Seoul, Republic of Korea; 2 Library of Marine Samples, Korea Institute of Ocean Science & Technology, Geoje, Republic of Korea; 3 Marine Ecosystem and Biological Research Center, Korea Institute of Ocean Science & Technology, Ansan, Republic of Korea; Stazione Zoologica Anton Dohrn, ITALY

## Abstract

The marine diatoms *Thalassiosira proschkinae* and *T*. *spinulata* are relatively small in size; their taxonomic identities have been debated owing to the diverse morphological variations. In the present study, we isolated both morphotypes from Korean coastal waters and examined their fine structures and conducted molecular sequence comparisons. The morphological and molecular analyses showed that *T*. *proschkinae* and *T*. *spinulata* were certainly distinct, and phenotypic plasticity of valve structure was not noted. Based on the morphological similarity and phylogenetic relationship, we transferred *T*. *proschkinae* and *T*. *spinulata* to another genus *Minidiscus* within Thalassiosirales that includes small-sized species and proposed new combination names, *Minidiscus proschkinae* (Makarova) Park & Lee comb. nov. and *Minidiscus spinulatus* (Takano) Park & Lee comb. nov., respectively. The genus description of *Minidiscus* was emended.

## Introduction

*Thalassiosira* Cleve is one of the most speciose marine diatom genera, with over 180 species being listed by Algaebase [1]. Many species having the two known types of processes (fultoportula and rimoportula) have been newly described as belonging to the genus *Thalassiosira*, and the genus concept was gradually expanded with the highly diverse characters of new species. The broad concept of characters of the species belonging to the genus *Thalassiosira* has hindered the determination of the inter-specific relationships, and recent phylogenetic studies showed para- or polyphyletic relationships that was consisted of 10 different lineages within in the order Thalassiosirales [2–4]. To construct the phylogenetic classification of the order Thalassiosirales, some authors attempted to separate the ambiguous species of *Thalassiosira* into new genera such as *Shionodiscus* Alverson, Kang et Theriot [5] and *Conticribra* Stachura-Suchoples et Williams [6], or transfer some doubtful species such as *Thalassiosira constricta* Gaarder into the phylogenetically appropriate genus *Bacterosira* Gran [7]. Despite these efforts, this order still contains phylogenetically ambiguous taxa such as *Thalassiosira*, the phylogenetic position of the species of which needs to be determined.

Of the members of *Thalassiosira*, the small-sized species *T*. *proschkinae* Makarova was originally described from the Sea of Azov on August 24, 1962 by Makarova [8]; another small-sized species *T spinulata* Takano was first described from the Japanese waters by Takano [9]. Both the species shared morphological characters such as a small size of less than 10 μm, the presence of a rimoportula on the valve face, one central fultoportula, and one ring of marginal fultoportulae. Makarova [10] suggested that *T*. *spinulata* was conspecific with *T*. *proschkinae* and reduced the species as a variety of *T*. *proschkinae*, namely, *T*. *proschkinae* var. *spinulata* (Takano) Makarova. Hasle & Syvertsen [11] also agreed to Makarova’s consideration and mentioned that the two species could not be distinguished by light microscopy. However, the taxonomic relationship between the species has been clarified by the differences of areola structure and valve silicification [12–14]. The taxonomic relationship between *T*. *proschkinae* and *T*. *spinulata* regarding whether the areola structure is an intra-specific phenotypic variation or inter-specific distinguishing character needs to be determined [13].

In *Thalassiosira*, the rimoportula could be positioned either on the valve face or the valve margin. Hasle [15] divided the genus *Thalassiosira* into two groups based on the positions of rimoportula. Hasle & Syvertsen [11] improved the original subgrouping proposed by Hasle [15]: Subgroup A is characterized by the presence of a rimoportula near the valve mantle, usually with an external extension; Subgroup B has a rimoportula located on the valve face without external extensions. Alverson et al. [5] transferred 26 *Thalassiosira* species in subgroup B into the genus *Shionodiscus*, which is characterized by the rimoportula on the valve face and no extension of the processes externally. Since *Thalassiosira* species in subgroup B were transferred to the genus *Shionodiscus*, most *Thalassiosira* species have a rimoportula at the valve margin [14]. In addition to *Shionodiscus*, some genera have the rimoportula on the valve face in Thalassiosirales, namely, *Minidiscus* Hasle, *Livingstonia* Prasad, and *Skeletonema* Greville. The position of rimoportula on the valve face in *T*. *proschkinae* and *T*. *spinulata* hinder the identification of their phylogenetic position in the order Thalassiosirales.

In the present study, we clarified the taxonomic identities between *T*. *proschkinae* and *T*. *spinulata* based on the fine surface structures and DNA sequence similarities and investigated the phylogenetic position of the tested small *Thalassiosira* species within the Thalassiosirales lineage by conducting molecular comparisons. The fine structure and molecular sequence analyses revealed the phylogenetic relationship of the two *Thalassiosira* species leading to a reconsideration of the order Thalassiosirales.

## Materials and methods

### Ethics statement

The field studies did not involve endangered or protected species, therefore, no specific permission were required for the sampled locations and activities.

### Environmental samplings and culture

**Fig 1:**
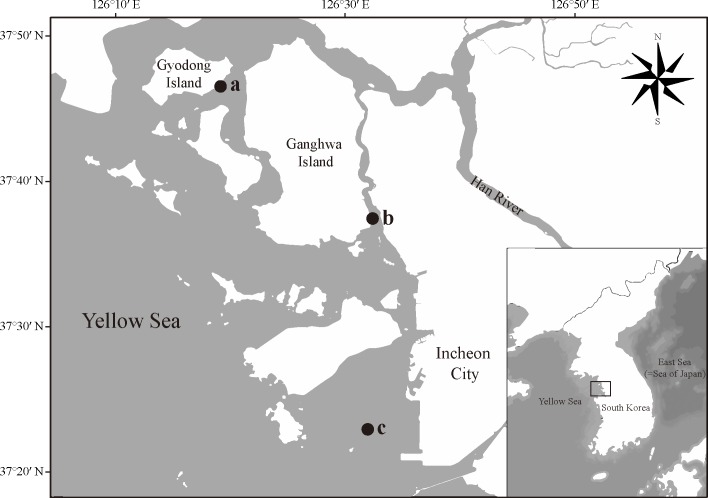
A map showing the sampling sites in Korea. The small letters beside the black dots indicated the collecting sites where *T*. *proschkinae* and *T*. *spinulata* strains. *T*. *spinulata* SMDC303 (a), *T*. *proschkinae* SMDC305 (b), *T*. *spinulata* SMDC050 (c).

Phytoplankton samples were collected from the province of Incheon in Korea between 2007 and 2016 by using a 20-μm mesh net by horizontal and/or vertical towing. The seawater had a salinity of <30 psu and contained many inorganic sediments because the areas investigated are affected by the influx of freshwater from the Han River through the major channels as well as movement of seawater by tides. Diatom cells were isolated using the capillary method and transferred to 24-well culture plates containing the collected and sterilized seawater amended with f/2 nutrients. Cells were incubated at 15°C under 12:12h L:D, with an irradiance of approximately 30 μmol photons·m^-2^·s^-1^. When the cell density reached approximately 10,000 cells·ml^-1^, 2 ml of each isolate was transferred and maintained in a 250 ml Erlenmeyer flask containing 100 ml seawater with f/2 nutrients. The following *Thalassiosira* species were isolated (Fig 1): *T*. *spinulata* SMDC050 from the Incheon coast (37°23ʹ02.46ʺN, 126°31ʹ59.48ʺE) on Jul 27, 2007; *T*. *spinulata* SMDC303 from the coast at Gyodong Island (37°46′25.69″N, 126°19′02.27″E) on Jun 13, 2016; and *T*. *proschkinae* SMDC305 from the coast at Ganghwa Island (37°37′32.09″N 126°32′22.79″E) on Jun 15, 2016.

### Morphological observations

Materials for scanning electron microscopy (SEM) were prepared for observing the intact and cleaned cells in two ways. The intact morphology from the isolates was observed by filtering live cells on a 3-μm polycarbonate membrane and washed with distilled water without any oxidation. The cleaned external and internal structures after the detachment of the organic matter were observed by adding some live cells to an equal volume of H_2_O_2_ and exposing them to ultraviolet light (312 nm) for 3 h on the UV-transilluminator (WUV-L50; witeg, Wertheim, Germany) and then washing them with distilled water. The cleaned cells were filtered and dried on a 3-μm polycarbonate membrane. The dried polycarbonate membranes were attached to aluminum stubs by using carbon conductive adhesive tapes and coated with 10 nm gold in an automatic sputter coater (Agar Scientific Ltd., Essex, UK). The prepared specimens were examined using FE-SEM (JSM7600F; Jeol, Tokyo, Japan) operating at 10 kV and 8 mm working distance. Valve dimensions from the SEM photographs were measured using ImageJ 1.51 software [16]. The terminology used for the structures discussed herein follows that of Ross et al. [17], Round et al. [18] and Theriot & Serieyssol [19].

### DNA analyses and phylogeny

Cultured samples of the three strains were harvested during exponential growth by using a series of centrifugation steps and stored at -70°C until the genomic DNA was extracted. The genomic DNA was extracted from cell pellets by using a DNeasy® Plant Mini Kit (Qiagen, Hilden, Germany). Nuclear rDNA fragments [i.e., nearly full-length small subunit ribosomal DNA (*SSU* rDNA) gene and the D1–D3 region of the large subunit ribosomal DNA (*LSU* rDNA)] and plastid-encoding genes (*psb*C and *rbc*L) were amplified and sequenced following protocols outlined in Alverson et al. [3] and Park & Lee [20]. Newly generated DNA sequences are available from GenBank using accession numbers KY912618, KY912621, KY912624, and KY912625 for *T*. *proschkinae* SMDC305; KY912616, KY912619, KY912622, and KY912626 for *T*. *spinulata* SMDC050; and KY912617, KY912620, KY912623, and KY912627 for *T*. *spinulata* SMDC303. These sequences were manually aligned to multiple sequence alignments containing 68 other broadly sampled species of Thalassiosirales [3, 7] (The alignment sequences file was provided as a phylip format in S1 File). A maximum likelihood phylogeny was inferred using RAxML ver. 8.0.20 by using the “-f a” option by performing 5,000 bootstrap replicates and inferring the best scoring tree [21]. Separate GTR+G models were applied to the *SSU* rDNA partition, *LSU* rDNA partition, the first + second codon positions for the combined *psbC* and *rbcL* genes, and the third codon positions for the combined *psbC* and *rbcL* genes.

## Results

### Morphology of *Thalassiosira proschkinae* SMDC305

**Fig 2:**
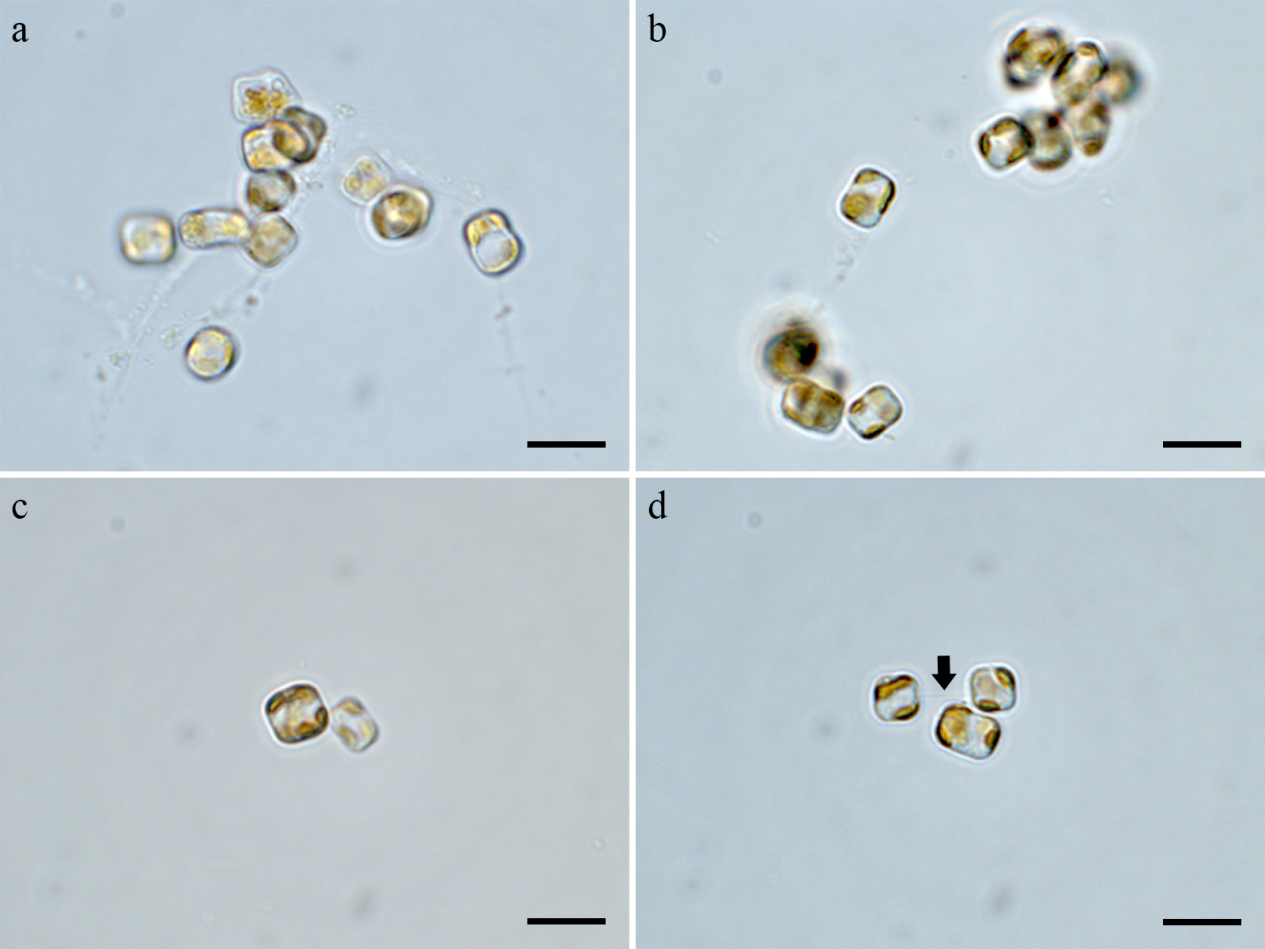
Light microscopy of *Thalassiosira proschkinae* SMDC305. (a, b) Aggregated cells. (c) Solitary cell. (d) Chain colony of two cells by mucilage thread (arrow) extruded from central fultoportula. Scale bars are 10 μm.

**Fig 3:**
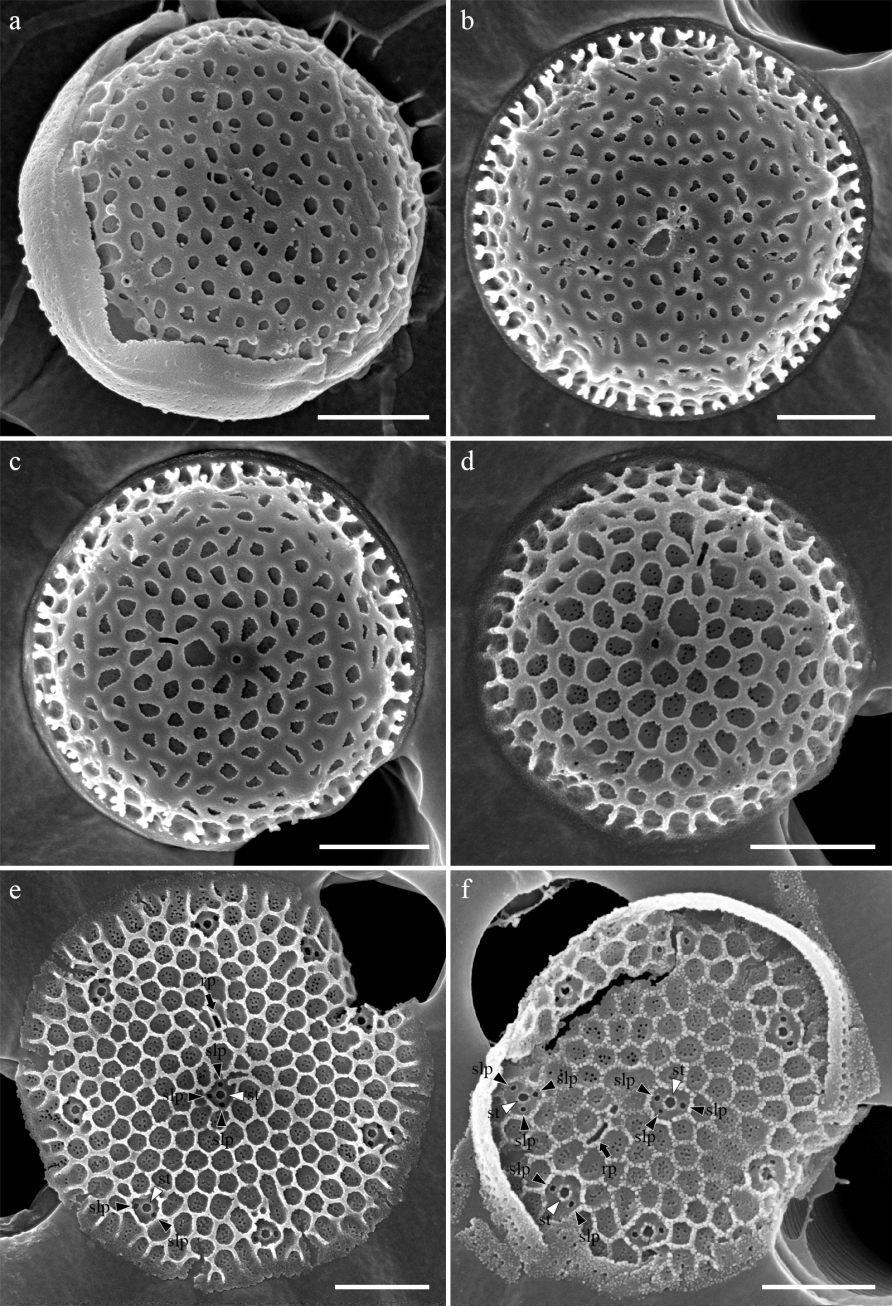
External whole valve of *Thalassiosira proschkinae* SMDC305. (a–d) Heavily silicified valves. (e, f) Valve cleaned by acid treatment showing the external opening of rimoportula (rp, black arrow) and internal structure of strutted tube (st, white arrowheads) surrounded by satellite pores (slp, black arrowheads). Scale bars are 1 μm.

**Fig 4:**
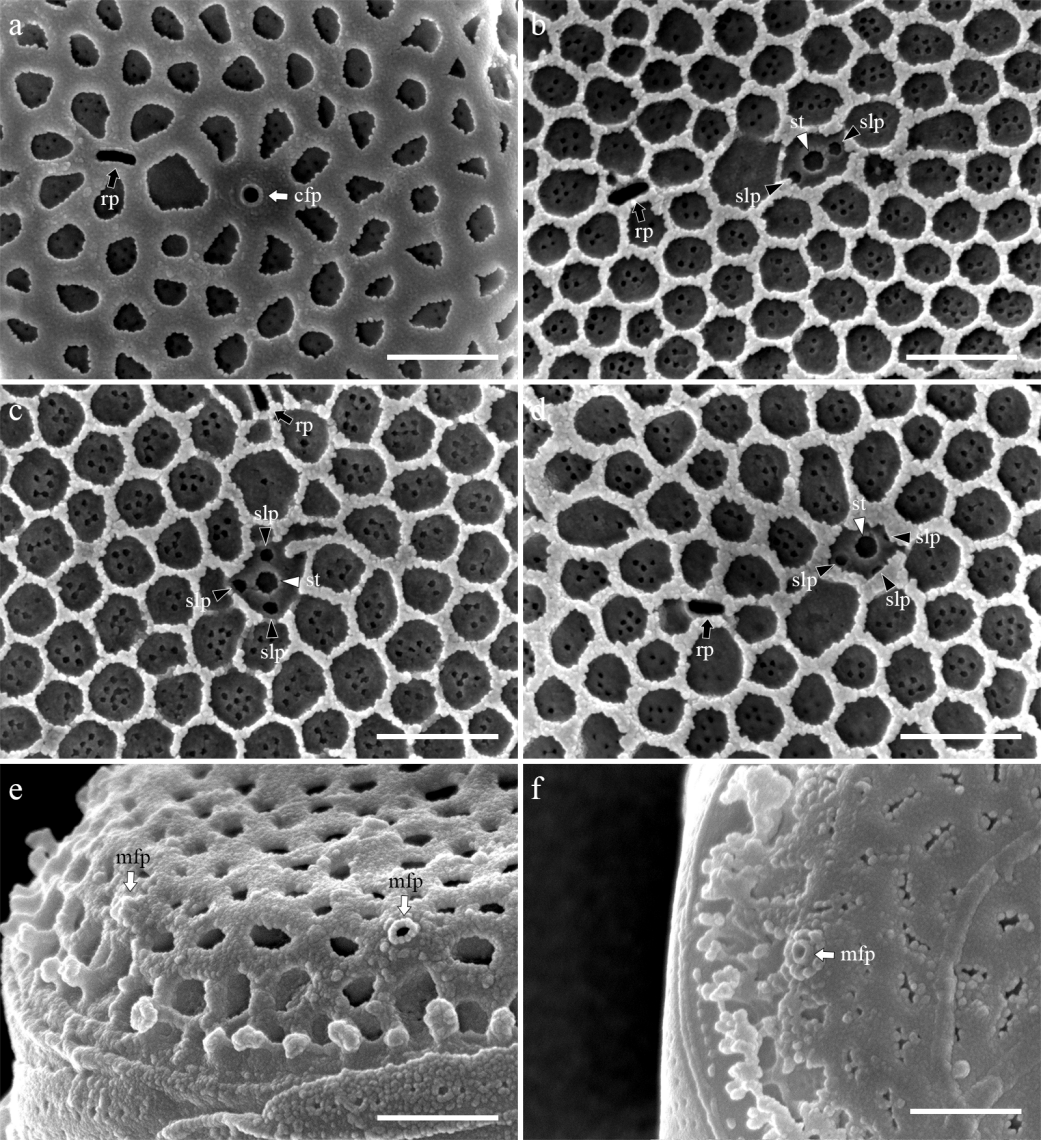
External valve view of *Thalassiosira proschkinae* SMDC305. (a) External small tube of central fultoportula (cfp, white arrow) and elliptic opening of rimoportula (rp, black arrow). (b–d) Acid cleaned valves showing the elliptical opening of rimoportula (rp, black arrow) and the exposed internal structure of strutted tube (st, white arrowhead) surrounded by two to three satellite pores (slp, black arrowheads). (e, f) External small tube of marginal fultoportula (mfp, white arrows) at the junction of valve face and mantle. Scale bars are 1 μm.

**Fig 5:**
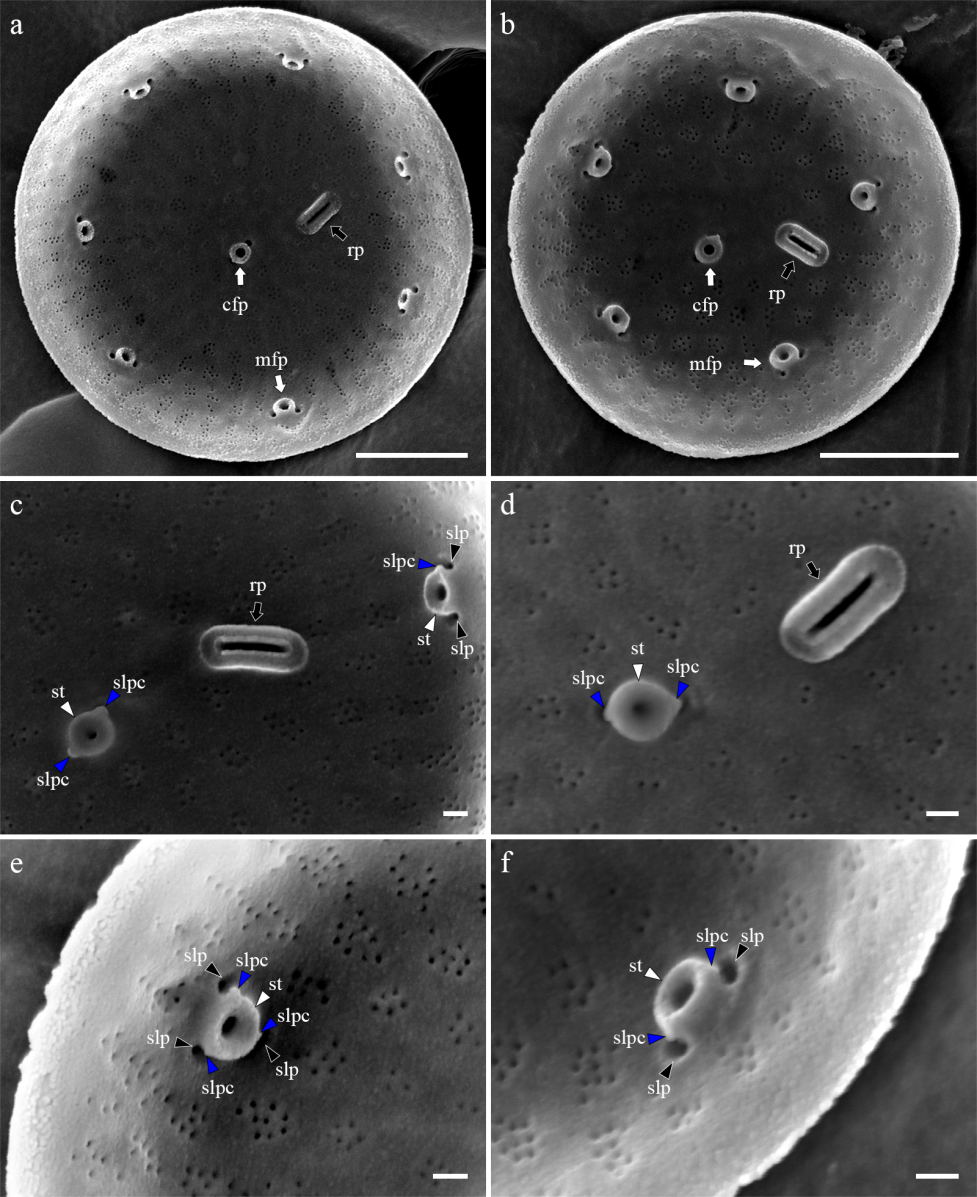
Internal valve view of *Thalassiosira proschkinae* SMDC305. (a, b) Internal whole valve having the central fultoportula (cfp, white arrow), one ring of marginal fultoportula (mfp, white arrow), and radially sessile rimoportula (rp, black arrow). (c) Rimoportula (rp, black arrow) between the central (cfp) and marginal fultoportula (mfp). Each fultoportula consist of strutted tube (st, white arrowhead), satellite pore cover (slpc, blue arrowheads), and satellite pore (slp, black arrowheads). (d) Central strutted tube (st, white arrows) with two satellite pore covers (slpc, blue arrowheads) and elliptical donut-shaped rimoportula (rp, black arrow). (e, f) Marginal fultoportula consist of strutted tube (st, white arrowhead), satellite pore covers (slpc, blue arrowheads), and satellite pores (slp, black aorrwheads). Scale bars are 1 μm.

Cells were aggregated (Fig 2A and 2B), solitary (Fig 2B and 2C) or forming 2–3 cells chain colonies by mucilage thread extruded from central fultoportula (Fig 2D). Valves were circular with a flattened valve face and sharp-angled mantle, 2.46–8.46 μm in diameter. Areolation was linear to sub-linear (Fig 3A–3F). Areolae were loculate and structured by external foramen and internal cribrum. The foramina were an irregular circular to obtuse angled polygonal structures (Fig 4A–4F), and the cribra consisted of several tiny pores (Fig 5A–5F). A single fultoportula was situated at the center of the valve (Fig 3A–3F), and it was structured as a small tube externally surrounded by silica granules (Fig 3A–3C, 4A); the strutted tube was internally surrounded by two to three satellite pores (Figs 4B–4D and 5A–5D). One ring of marginal fultoportulae was situated in the junction between the valve face and mantle (Fig 4E and 4F). In all, 6–8 marginal fultoportulae were present, with distances of 1.24–1.91 μm among them. The external openings of the marginal fultoportulae were similar to the central fultoportula (Fig 4E and 4F), and the strutted tube was internally surrounded by two satellite pores (Figs 3E, 3F, 5A and 5F), often three (Fig 5B and 5E). All fultoportulae had tiny triangular-shaped satellite pores covered with slightly raised cowling internally (Fig 5C–5F). A single rimoportula was located on the valve face adjacent to the central fultoportula, and one large areola was present between the central fultoportula and the rimoportula (Fig 4A–4D). The external opening of the rimoportula varied in forms and was elliptic (Fig 4A–4D) and was occasionally not distinguished from the adjacent areolae (Fig 3A and 3B). Internally, the rimoportula was positioned on the basal silica wall, without any stalk (Fig 5A–5D). The slit of rimoportula was thin and straight like the external opening (Fig 5A–5D). The slit was surrounded by the heavily silicified elliptic rim (Fig 5C and 5D). The internal shape of the rimoportula resembled an ellipsoidal doughnut (Fig 5C and 5D).

### Morphology of *Thalassiosira spinulata* SMDC303

**Fig 6:**
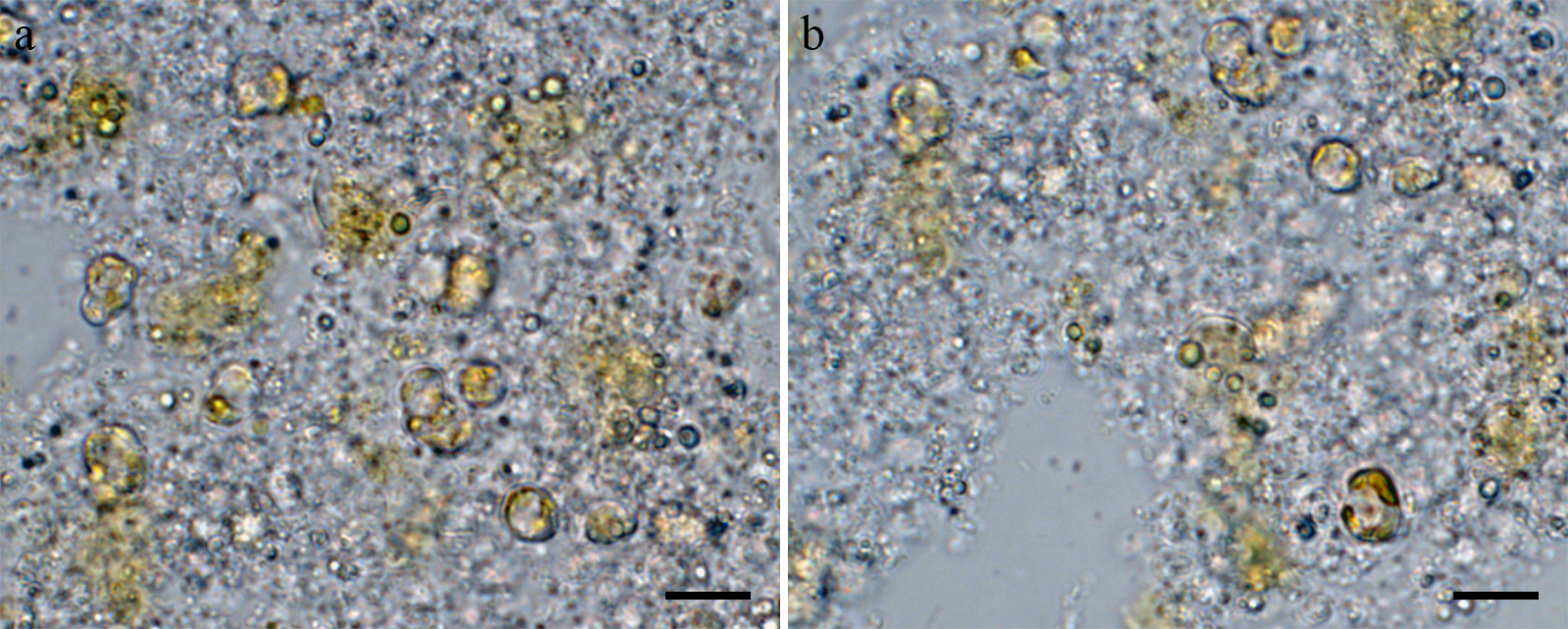
Light microscopy of *Thalassiosira spinulata* SMDC303. (a, b) Cells embedded by mucilage. Scale bars are 10 μm.

**Fig 7:**
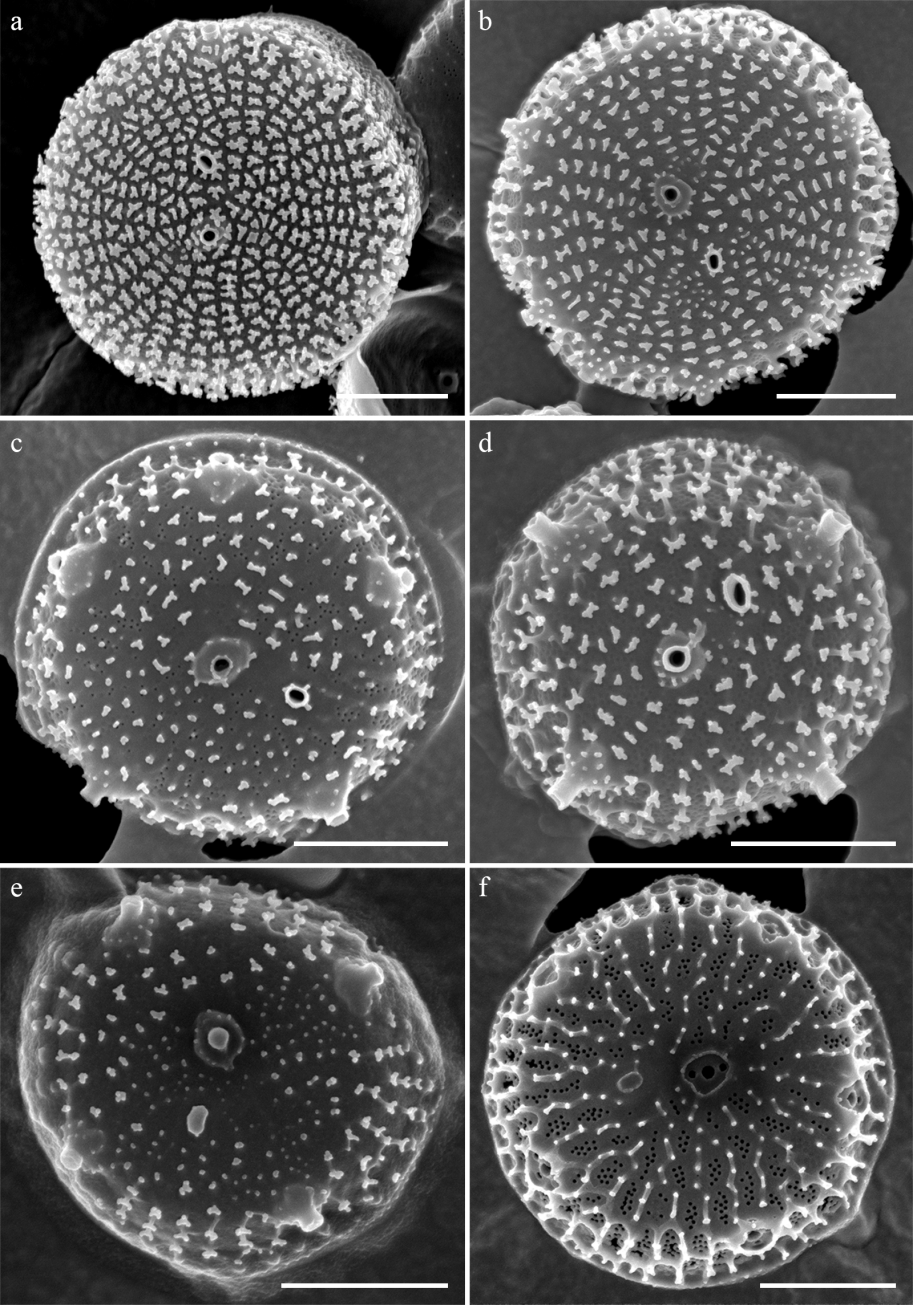
External whole valve of *Thalassiosira spinulata* SMDC303. (a, b) Heavily silicified valve. (c, d) Moderately silicified valve. (e, f) Poorly developed ribs. Scale bars are 1 μm.

**Fig 8:**
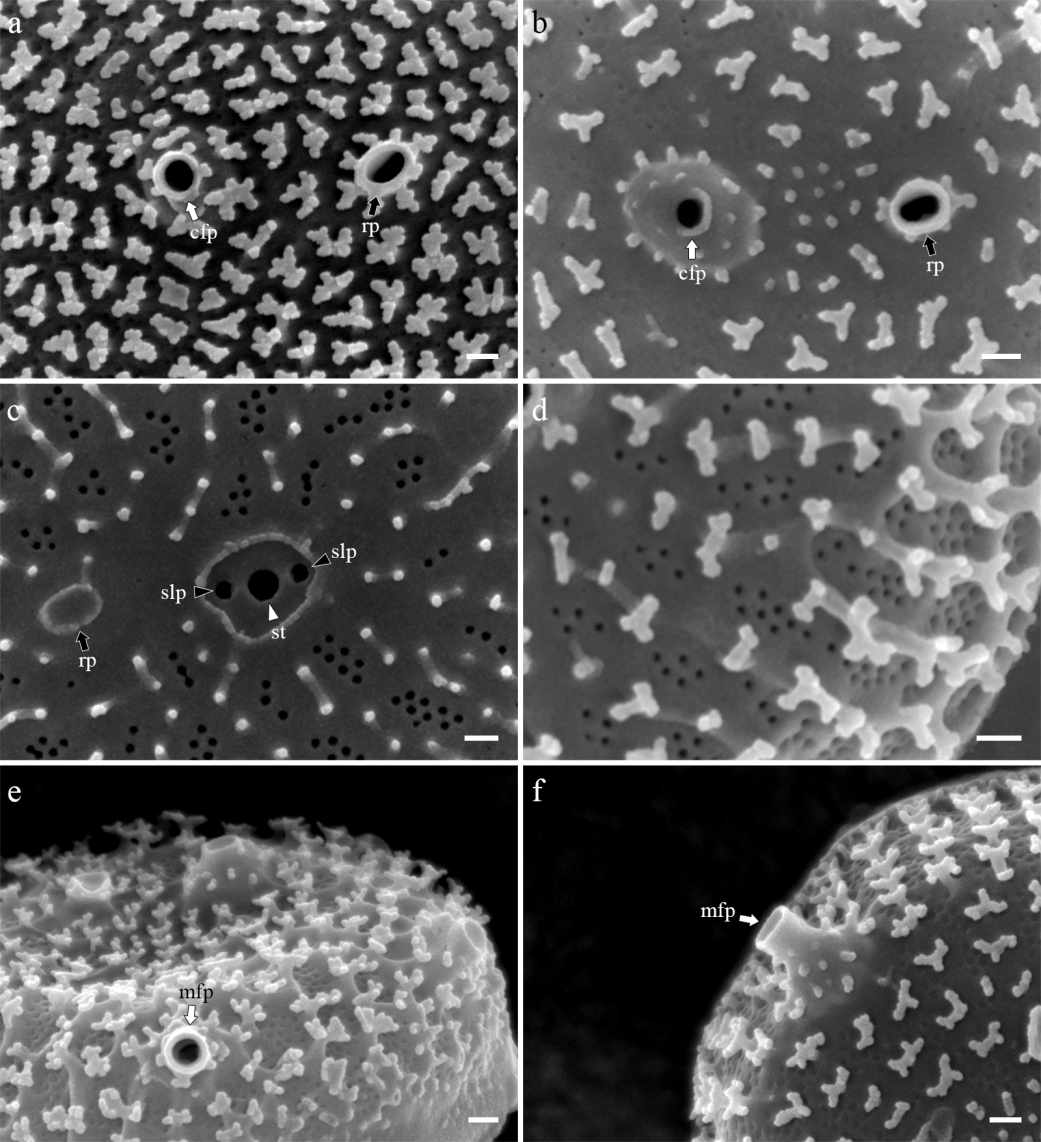
External valve view of *Thalassiosira spinulata* SMDC303. (a, b). External opening of central fultoportula (cfp, white arrow) and rimoportula (rp, black arrow). (c) Superimposed internal structure of central fultoportula consist of strutted tube (st, white arrowhead) and two satellite pores (slp, black arrows). (d) Y-shaped development of ribs between the areolae in the valve margin. (e) Circular opening of marginal fultoportula (mfp, white arrow). (f) Obtuse external tube of marginal fultoportula (mfp, white arrow). Scale bars are 0.1 μm.

**Fig 9:**
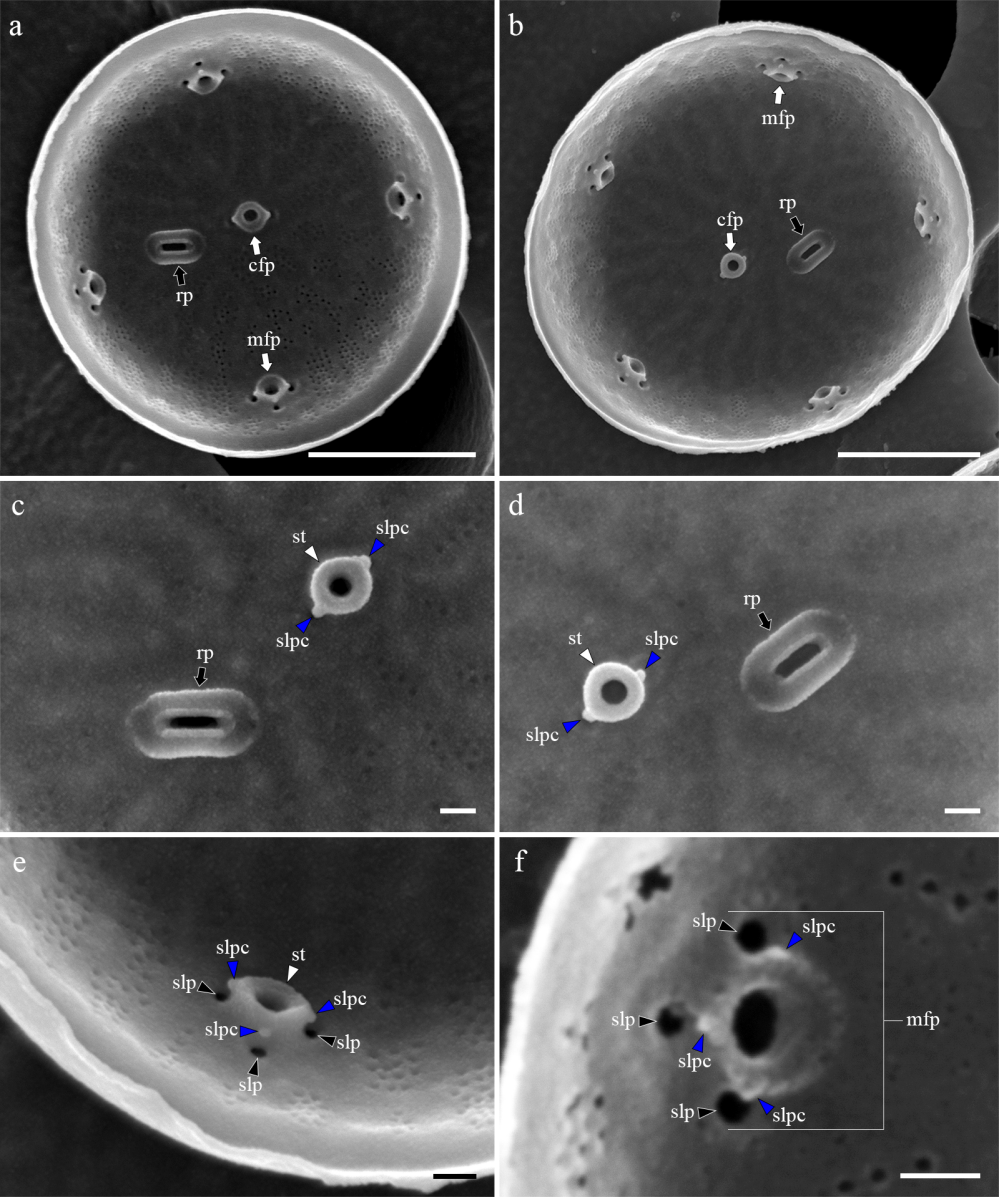
Internal valve view of *Thalassiosira spinulata* SMDC303. (a, b) Internal whole valve having the central fultoportula (cfp, white arrow), one ring of marginal fultoportula (mfp, white arrow), and radially sessile rimoportula (rp, black arrow). (c, d) Central strutted tube (st, white arrowhead) with two satellite pore covers (slpc, blue arrows), and elliptical donut-shaped rimoportula (rp, black arrow). (e, f) Marginal fultoportula consist of strutted tube (st, white arrowhead), satellite pore covers (slpc, blue arrows) and satellite pores (slp, black arrowheads). Scale bars are 1 μm for Fig 6A and 6B and 0.1 μm for Fig 6C–6F.

Cells were embedded in a mucilage (Fig 6A and 6B), Valves were circular with a flat valve face and obtuse-angled mantle and were 2.53–5.04 μm in diameter. Areolae almost incomplete in most cells and were neither loculate nor poroid (Fig 7A–7F). No true foramen was found externally (Fig 8A and 8B), and the cribra was radially continuous internally and occasionally naked externally (Fig 8C and 8D). Instead of the areolae, the valve face contained granules that were situated on the valve (Figs 7A–7F and 8A–8C), whereas Y-shaped ribs were observed on the valve margin (Fig 8D). A single fultoportula was situated at the center or subcenter of the valve (Fig 7A–7F) and it was structured externally as a stumpy tube surrounded by a hyaline area (Figs 7B–7E and 8B), and the strutted tube was surrounded internally by two satellite pores (Fig 9A–9S). One ring of marginal fultoportulae was situated in the junction between the valve face and valve mantle (Fig 8E and 8F). In all, 4–10 marginal fultoportulae were present, with distances of 1.11–1.93 μm between pairs of them. The external openings were similar to the central fultoportula (Fig 8E and 8F), but the strutted tube was internally surrounded by four satellite pores (Fig 9E and 9F). The satellite pore covers were semi-circular (Fig 9C and 9D) and attached near the tips of the strutted tube away from the satellite pores (Fig 9E and 9F). A single rimoportula was situated on the valve face adjacent to the central fultoportula (Fig 9A–9D). The external opening of the rimoportula was a sub-circular to elliptical tube, and it was smaller than the external tube of the central fultoportula (Fig 8A and 8B). Internally, the rimoportula was positioned on the basal silica wall, without any stalk (Fig 9A–9D). The internal opening was always elliptical, and the heavily silicified rim was located on the opening, like the external opening (Fig 9C and 9D). The internal shape of the rimoportula resembled an ellipsoidal doughnut (Fig 9C and 9D).

### Sequence comparison and phylogenetic position of *T*. *proschkinae* and *T*. *spinulata*

The intra- and interspecific differences of the molecular sequences between *T*. *spinulata* and *T*. *proschkinae* are shown in Table 1. The intraspecific differences of *T*. *spinulata* were calculated from the two strains, SMDC050 and SMDC305, but the intraspecific differences of *T*. *proschkinae* could not be calculated because only one strain of *T*. *proschkinae* was obtained. The intraspecific divergences of *T*. *spinulata* did not show any differences among the four markers. However, the interspecific divergences between *T*. *spinulata* and *T*. *proschkinae* showed various differences according to the markers. The SSU rRNA of both species were nearly identical; there was only 1 nucleotide difference in the 1,708 bp, and the D1–D3 region of the LSU rRNA differed by 6 bp of 825 bp. The nucleotide differences in the two chloroplast genes were higher than those in the nuclear rRNA markers: the psbC by 19 bp of 1,129 bp and the rbcL by 21 bp of 1,419 bp.

**Table 1:** Intraspecific genetic distances between the two strains of *T*. *spinulata*, and interspecific genetic distances between *T*. *proschkinae* and *T*. *spinulata* for the two nuclear rRNA and two chloroplast genes.

Strains	18S	28S D1–D3	*psb*C	*rbc*L
*T*. *spinulata* SMDC303 vs. *T*. *spinulata* SMDC050	0.0000	0.0000	0.0000	0.0000
*T*. *spinulata* SMDC303 vs. *T*. *proschkinae* SMDC305	0.0006 (1707/1708)	0.0074 (819/825)	0.0120 (1398/1415)	0.0122 (1110/1129)
*T*. *spinulata* SMDC050 vs. *T*. *proschkinae* SMDC305	0.0006 (1646/1647)	0.0074 (819/825)	0.0120 (1398/1415)	0.0122 (1110/1129)

**Fig 10:**
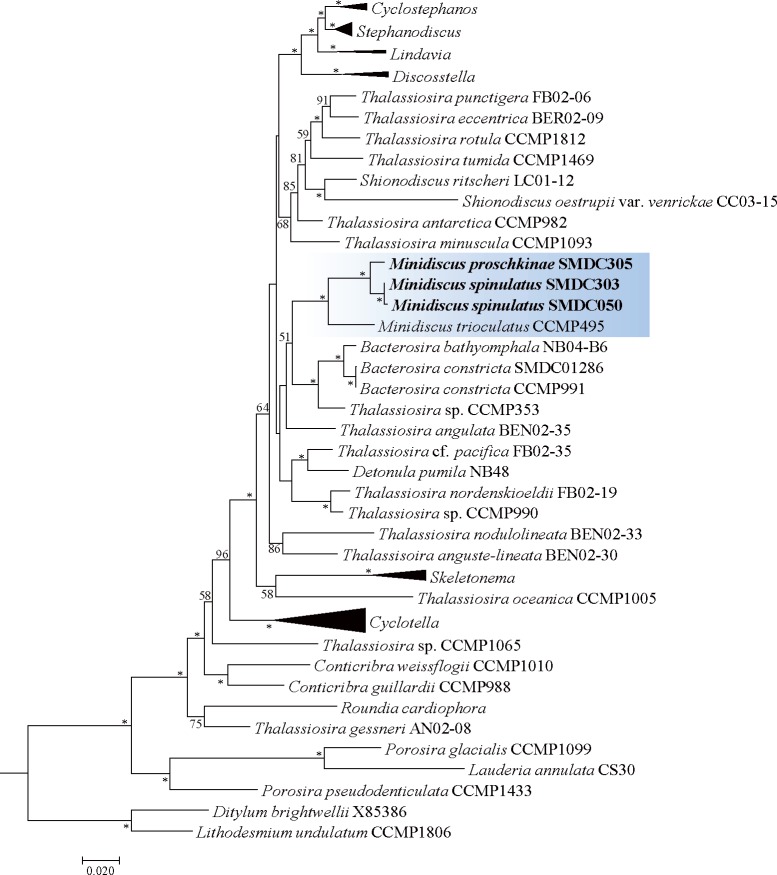
A phylogenetic tree for Thalassiosirales inferred from the maximum likelihood (ML) by using RAxML. Bootstrap values of ≥50% are shown, and nodes supported by bootstrap values of ≥95% are marked with an asterisk (*).

Multi-gene phylogenetic analysis strongly supported a relationship between *T*. *proschkinae* and *T*. *spinulata*. In addition, the phylogeny recovered a strongly supported sister relationship between the two small *Thalassiosira* species and *Minidiscus trioculatus* (Fig 10). This clade was related to the genus *Bacterosira* with moderate support (Fig 10).

## Discussion

### Clarifying the taxonomic relationship between *T*. *proschkinae* and *T*. *spinulata*

#### Morphological comparison

**Fig 11:**
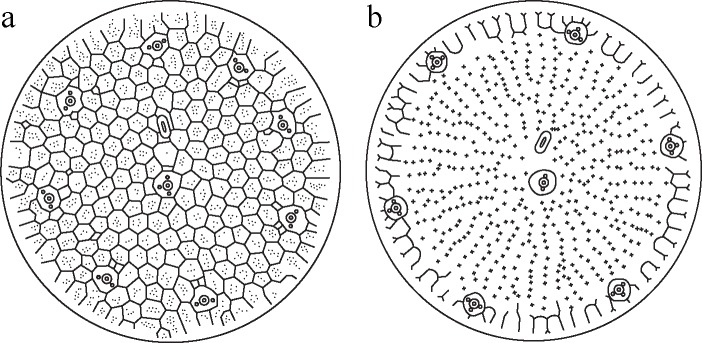
Diagram of two small *Thalassiosira* species. (a) *T*. *proschkinae*. (b) *T*. *spinulata*.

Certain morphological differences were observed in the two isolated morphotypes, *T*. *proschkinae*/*T*. *spinulata* in Korea. The two morphotypes showed differences in the external valve ornamentation, internal cribra, external opening of rimoportula and fultoportula, and the arrangement of satellite pores of marginal fultoportula (Fig 11). In particular, the external valve ornamentation and the arrangement of satellite pores of marginal fultoportulae were the critical characteristics to distinguish both the morphotypes, and we suggest that *T*. *proschkinae* and *T*. *spinulata* are distinct species.

The areola structure of *Thalassiosira* species was traditionally characterized by the external foramen and internal cribra, and it was re-divided by the foramen shapes and the cribra [11, 22]. The foramen was divided into two types, loculate and poroid, by the level of covering of the roof of areola [11]. *T*. *proschkinae* consistently showed a distinct loculate areola structure that consists of the internally individual cribra and externally irregularly shaped foramen, whereas *T*. *spinulata* does not have a true areola, and the internally continuous cribra structure was exposed to the valve exterior. Although the areola structure showed variability according to the level of silicification, for instance, *T*. *gravida*/*T*. *rotula* complex [23], the areola structure of *T*. *proschkinae* and *T*. *spinulata* never showed the intermediate developing status in the present study. Martín-Cereceda & Cox [13] investigated the development of the valve structure of *T*. *spinulata* according to the changes in salinity and silicic acid concentration. They also did not observe mature areolae in *T*. *spinulata*. Therefore, the absence of the true areola is the species-specific character for *T*. *spinulata*, and thus *T*. *proschkinae* and *T*. *spinulata* are morphologically distinct species.

Two types of portula, rimoportula and fultoportula, are structured by the penetration of the base silica layer and can be easily distinguished by the complex internal structure. However, the external structure of the two portula is somewhat simple and expressed by pore- and tube-like structures [2]. The external structure of the two portula is the diagnostic characteristic for distinguishing the two small-sized *Thalassiosira* species. The external opening of rimoportula in *T*. *proschkinae* is a pore-like opening and situated between the loculate areolae, whereas that in *T*. *spinulata* is a conspicuous tube. The marginal fultoportula of the two small-sized *Thalassiosira* species have a tube-like opening, but the size of the external tube is different: the external tube of *T*. *proschkinae* is small and inconspicuous, whereas that of *T*. *spinulata* is large and remarkable.

The satellite pore of the marginal fultoportulae was a fairly stable morphological character in the order Thalassiosirales [19]. In the present study, *T*. *spinulata* had three satellite pores of marginal fultoportulae, it agreed to the previous description [14]. However, *T*. *proschkinae* had two satellite pores in marginal fultoportula and sometimes rarely three reported by Park et al. [14]. Despite of the number of satellite pore in marginal fultoportula in the thalassiosiroid diatoms is a stable characteristic, this structural variation in *T*. *proschkinae* is unusual and provided the additional morphological evidence to separate *T*. *proschkinae* from *T*. *spinulata* that had three satellite pores in marginal fultoportula continuously. When *T*. *proschkinae* had three satellite in marginal fultoportula like *T*. *spinulata*, it can be distinguished by the pore position around the strutted tube. Of the three satellite pores, two were parallel along the valve margin, and one was located differently: in *T*. *proschkinae*, the pore was positioned towards the valve face behind the strutted tube; in *T*. *spinulata*, it was located towards the valve mantle. When the valves were observed in the valve plane without any tilts, *T*. *proschkinae* seemed to have two satellites because the other pore was hidden behind the strutted tube, whereas, in *T*. *spinulata*, all three satellite pores were visible. The number and position of satellite pores of marginal fultoportula supported two species are morphological distinct.

#### Global distribution of the two species

**Fig 12:**
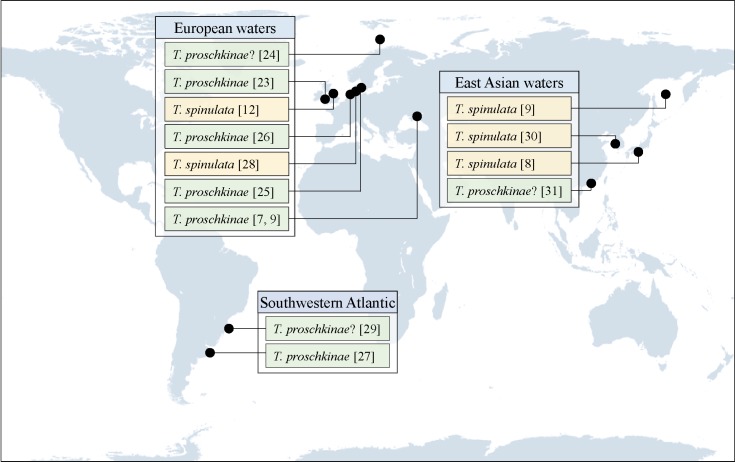
Biogeography of *T*. *proschkinae* and *T*. *spinulata* from 14 literature reports and the present study.

Since Makarova [10] suggested that *T*. *spinulata* was ranked on various *T*. *proschkinae*, most records worldwide for both the species have been mainly reported as *T*. *proschkinae*. Based on the above-mentioned morphological differences between *T*. *proschkinae* and *T*. *spinulata*, we re-identified the previous records on both the species (Table 2) and confirmed their distribution (Fig 12). Both the species have mainly been reported from the European waters, East Asian waters, and Southwestern Atlantic waters (Fig 12). Among the 14 records of *T*. *proschkinae* and *T*. *spinulata*, most records have reported *T*. *proschkinae*, but *T*. *spinulata* has also been reported from the areas where *T*. *proschkinae* occurred. Both the species have been mainly found from the estuaries characterized by low salinity (<30 psu) and high turbulence (Table 2). Although there were no descriptions regarding the co-occurrence of the two morphotypes simultaneously in the same area, we simultaneously found both the species in the estuaries of Korea. The co-occurrence of both the species indicated that they share habitat features and might have recently evolved as a sympatric speciation.

**Table 2:** Literatures on *T*. *proschkinae/T*. *spinulata* and re-identification based on the morphological criteria and their distribution.

Literatures	Previous identification	Our interpretation	Distribution
Makarova et al. [8] (p. 922, pl. 1, fig 1–7)	*T*. *proschkinae*	*T*. *proschkinae*	Sea of Azov
Takano [9] (p. 33, figs 1B, 14–25)	*T*. *spinulata*	*T*. *spinulata*	Japanese coasts (Dokai Bay, Atsumi Bay, downstream of Smida River, Hamanako Lake)
Belcher & Swale [24] (p. 141, fig. 3)	*T*. *proschkinae*	*T*. *proschkinae*	British coasts
Makarova [10] (p. 80, pl. 51, figs 13–22)	*T*. *proschkinae*	*T*. *proschkinae*	Azov and Caspian Seas
	*T*. *proschkinae* var. *spinulata*	*T*. *spinulata*	Okhotsk Sea
Metzeltin & Witkowski, [25] (pl. 43)	*T*. *proschkinae*	uncertain (only LM micrographs)	Bear Island (North Atlantic)
Feibicke et al. [26] (p. 159, pls I and II)	*T*. *proschkinae*	*T*. *proschkinae*	The Schlei fjord of the Baltic Sea in the north of Germany
Muylaert & Sabbe, [27] (p. 110, figs 24–26)	*T*. *proschkinae*	*T*. *proschkinae*	The estuaries of the Schelde (Netherland) and the Elbe (Germany)
Hasle & Syvertsen, [11] (p. 84, pl. 13, figs a–c)	*T*. *proschkinae*	*T*. *proschkinae*	cosmopolitan
Sar et al. [28] (p. 215, figs 49, 50)	*T*. *proschkinae*	*T*. *proschkinae*	Pinamar and Villa Gesell, Argentina
Hoppenrath et al. [29] (p. 281, fig. 48)	*T*. *proschkinae*	*T*. *spinulata*	Helgoland, Germany
Tremarin et al. [30] (p. 1106, figs 24–27)	*T*. *proschkinae*	uncertain (only LM micrographs)	Paranaguá River, Brazil
Park et al. [31] (p. 75, pl. V, figs 27–32)	*T*. *proschkinae*	*T*. *spinulata*	Korean coasts
Martín-Cereceda & Cox [13] (p. 565, figs 1–23)	*Thalassiosira* isolate	*T*. *spinulata*	British coast
Li et al. [32] (p.101, fig. 106)	*T*. *proschkinae*	*T*. *proschkinae*?	Zhelin Bay and Shantou off the Guangdong coast

### Comparison with morphologically similar taxa and identification of the phylogenetic position of the two small-sized *Thalassiosira* species

*T*. *proschkinae* and *T*. *spinulata* were characterized by a diameter of less than 10 μm, a rimoportula on valve face, and an internally ellipsoidal donut-shaped rimoportula. These morphological features were compared to the genera including small-sized *Thalassiosira* species having rimoportula on the valve face, such as *Minidiscus*, *Shionodiscus*, and *Livingstonia*.

The extremely small size of *T*. *proschkinae* and *T*. *spinulata* is a rare feature of some *Thalassiosira* species. Several species have less than 10 μm diameter: *T*. *mala* Takano, *T*. *profunda* (Hendey) Hasle, and *T*. *exigua* Fryxell & Hasle. When Takano [9] initially described *T*. *spinulata*, he already distinguished it from the small-sized *Thalassiosira* species by the rimoportula position. The *Thalassiosira* species that have the rimoportula on valve face were transferred to the genus *Shinodiscus* by Aleverson et al. [5], but several species (*T*. *ferelineata* Hasle & Fryxell, *T*. *ignota* Makarova, *T*. *intrannula* Herzig & Fryxell, *T*. *maculata* Fryxell & Johansen, and *T*. *rosulata* Takano) still remained in the genus *Thalassiosira*. These species differ from *T*. *proschkinae* and *T*. *spinulata* by larger size, no external extension of fultoportula, and the internally stalked rimoportula. Although further study is needed to confirm the taxonomic position of the species having the rimoportula on the valve face, this issue was not addressed in the present study and needs further investigation.

The genus *Minidiscus* is morphologically the most similar taxa to *T*. *proschkinae* and *T*. *spinulata*; it is characterized by species having diameter of less than 10 μm, a rimoportula on the valve face adjacent to a central fultoportula, and lack of a ring of marginal fultoportulae [18, 33]. *T*. *proschkinae* and *T*. *spinulata* share these morphological characters, as well as having an ellipsoidal doughnut-shaped rimoportula; however, the two small *Thalassiosira* species have a marginal ring of fultoportulae. Although the latter character does not coincide with the generic characteristics of *Minidiscus*, the positioning of the fultoportula in *Minidiscus* species is known to vary: *M*. *chilensis* Rivera has a fultoportula on the valve center [34]; *M*. *comicus* Takano has a fultoportula in midway between the valve center and margin [9]; and *M*. *trioculatus* has a varyingly positioned fultoportula depending on valve diameter [35]. Therefore, the absence of fultoportula in the valve margin of *Minidiscus* species represents a characteristic that differs among species and is not a generic characteristic. Except for the position of marginal fultoportula, *T*. *proschkinae* and *T*. *spinulata* are morphologically close to *Minidiscus*. In particular, the internally ellipsoidal-shaped rimoportula of *T*. *proschkinae* and *T*. *spinulata* is observed in all *Minidiscus* species.

*Livingstonia palatkaensis* Prasad is also characterized by a diameter of 10 μm, rimoportula on the valve face, and a ring of marginal fultoportulae [36] (Table 3). However, the absence of central fultoportula, the externally hood shape of marginal fultoportula, and the freshwater habitat of *L*. *palatkaensis* differentiates it from *T*. *proschkinae* and *T*. *spinulata*. Although Prasad & Nienow [36] did not compare *L*. *palatkaensis* with *Minidiscus* species, the shared characters between the two taxa is yet uncertain. The molecular sequence of *L*. *palatkaensis* might provide information to reveal the relationship between the two taxa.

**Table 3:** Comparison of the morphological characters of two small *Thalassiosira* species and the thalassiosiroid taxa having the rimoportula on valve face (*Minidiscus*, *Livingstonia palatkaensis*, and *Shionodiscus*) and *Thalassiosira nordenskioeldii* as the representative of the genus *Thalassiosira*.

Morphological characters	*Thalassiosira proschkinae*	*Thalassiosira spinulata*	*Minidiscus* spp.	*Livingstonia palatkaensis* (monotype)	*Shionodiscus* spp.	*Thalassiosira nordenskioeldii*(type species)
Diameter	2.5–8.5	2.5–5.0	less than 10 μm	2.5–6.0	more than 10 μm	10–50
External valve structure	loculate areolae	streusel-like silicification	hyaline to areolae	loculate areolae	loculate areola	loculate areolae
Internal valve structure (cribra type)	individual	continuous radially with one to two row of pores	individual, continuous, unperforated	individual	individual	individual
Position of RP	valve face	valve face	valve face	valve face	valve face	valve margin
External opening of RP	areola	rimmed pore without any extension	rimmed pore without any extension	rimmed pore without any extension	pore without any extension	tube-like extension
Internal shape of RP	ellipsoidal ring without any stalk	ellipsoidal ring without any stalk	ellipsoidal ring without any stalk	ellipsoidal ring without any stalk	fan-shaped on stalk	asymmetric labium on stalk
Number of CFP	one	one	one	absent	one to several	one
Position of MFP	on junction between valve face and mantle	on junction between valve face and mantle	valve face to sub-margin, and absence	on junction between valve face and mantle	mantle	mantle
External opening of MFP	rimmed pore buried by siliceous ribs	tube-like extension	tube-like extension	hyaline hood	pore without any extension	tube-like extension
Number of satellite pores of MFP	two (three)	three	two to three	two	three to four	four
References	this study	this study	Quiroga & Chrétiennot-Dinet [12], Aké-Castillo et al.[37], Kaczmarska et al. [12, 35, 38]	Prasad & Nienow [36]	Alverson et al. [5]	Hasle [39]

RP, rimoportula; CFP, central fultoportula; MFP, marginal fultoportula.

*Shionodiscus* also has a rimoportula on the valve face [5]. Although the position of rimoportula in *Shionodiscus* is similar to that in *T*. *proschkinae* and *T*. *spinulata*, the species belonging to this genus certainly differ from the two species by the internal structure of rimoportula, no fultoportula external extension without an outward extension, larger cell size, and external foramen shape [5] (Table 3). These features are the reason why *T*. *spinulata* and *T*. *proschkinae* remained in the genus *Thalassiosira*. Martín-Cereceda & Cox [13] presumed that *T*. *proschkinae* should probably be included in the genus *Shionodiscus*. Our morphological observation of *T*. *proschkinae* shows that the inclusion of *T*. *proschkinae* in *Shionodiscus* is not proper.

Multi-gene phylogeny for *T*. *proschkinae* and *T*. *spinulata* showed a close relationship to the other small-sized species, *Minidiscus trioculatus* that was phylogenetically related to *T*. *nordenskioeldii* and its allied species, *T*. *pacifica* and *Detonula pumila* in the previous phylogenetic analyses [4, 7]. With the addition of the sequence data of *T*. *proschkinae* and *T*. *spinulata*, the phylogenetic position of *M*. *trioculatus* is restructured as a sister clade to two small *Thalassiosira* species. Its relocation of *M*. *trioculatus* morphologically more proper than the relationship with *T*. *nordenskioeldii* and its allied species (Table 3). Taxon sampling in the phylogenetic analysis has emphasized as an important issue to improve the resolution towards the natural classification of diatom [40, 41]. The broad concept of the genus *Thalassiosira* still involves the ambiguous taxa as *Thalassiosira* [14]. Many *Thalassiosira* species are not assigned phylogenetic position, but their sequence data have not yet been analyzed. Recently, Park et al. [7] revised the genus *Bacterosira* by transferring it to *T*. *constricta*. Such studies from phylogenetically ambiguous species are needed to resolve the phylogenetic chaos of the order Thalassiosirales.

The morphological similarities and molecular sequences data between the two small-sized *Thalassiosira* and *Minidiscus* suggested that both the species should be transferred to *Minidiscus*. The genus *Minidiscus* was established to accommodate the species formerly known to *Coscinodiscus trioculatus* by Hasle [33]. She focused on the lack of the marginal fultoportulae and separated *C*. *trioculatus* from the genus *Thalassiosira*. Since the establishment of the genus, 10 *Minidiscus* species have been additionally described based on the genus concept by Hasle [33], namely, the valve size, fultoportula position on the valve, and hyaline margin. In addition, we noticed the similarity of rimoportula structure in *Minidiscus* species and it is proper to define the genus concept for *Minidiscus*. In conclusion, we transferred *T*. *proschkinae* and *T*. *spinulata* to the genus *Minidiscus* and renamed them *Minidiscus proschkinae* (Makarova) J.S. Park & J.H. Lee *comb*. *nov*. and *Minidiscus spinulatus* (Takano) J.S. Park & J.H. Lee *comb*. *nov*., respectively, and emended the description of the genus *Minidiscus*.

***Minidiscus proschkinae* (Makarova) comb. nov. J.S. Park *&* J.H. Lee.** Basionym: *Thalassiosira proschkinae* Makarova 1979, p. 922, pl. 1, Figs 1–7.

***Minidiscus spinulatus* (Takano) comb. nov. J.S. Park *&* J.H. Lee.** Basionym: *Thalassiosira spinulata* Takano 1981, p. 33, Fig 1B, 14–25. Synonym: *Thalassiosira proschkinae* var. *spinulata* (Takano) Makarova 1988

***Minidiscus* Hasle emended Park.** Cell in solitary, chain or clogged. Cell diameter less than 10 μm. Valve face flat to convex. Valve ornamentation various, loculate areolae, hyaline without any perforation, unlinked ribs. One central fultoportula; one to ten fultoportulae on valve face to valve margin. All fultoportulae externally opened with short tube and surrounded by siliceous hyaline. A single rimoportula adjacent to a central fultoportula, having an externally circular or elliptical small pore and an internally ellipsoidal ring of sessile lips.

## Supporting information

S1 File 71 multi-gene alignment sequences file. The multi-gene sequences of three strains, *T*. *proschkinae* SMDC305, *T*. *spinulata* SMDC050 and *T*. *spinulata* SMDC303, manually aligned based on the previous published 68 multi-gene alignment sequences [3, 7].
(PHY)
